# Involving End Users in the Development and Usability Testing of a Smartphone App Designed for Individuals With Prediabetes: Mixed-Methods Focus Group Study

**DOI:** 10.2196/59386

**Published:** 2025-02-11

**Authors:** Natalie Grieve, Kyra Braaten, Megan MacPherson, Sam Liu, Mary E Jung

**Affiliations:** 1 Faculty of Health and Social Development, University of British Columbia, 1238 Discovery Way, Kelowna, BC, V1V1V7, Canada, 1 250 807 9670; 2 Exercise Science, Physical and Health Education, University of Victoria, Victoria, BC, Canada

**Keywords:** usability evaluation, mHealth, usability testing, app, end-user, focus group, participant, survey, diabetes, user-centered, cognitive walkthrough, cognitive walkthroughs, questionnaire, mobile phone, digital health, prediabetes

## Abstract

**Background:**

Technology is more likely to be used when it is designed to meet the needs of end users. To supplement the Small Steps for Big Changes diabetes prevention program, a smartphone app was developed in partnership with past Small Steps for Big Changes clientele. Usability testing is critical for the ongoing use and adoption of mobile health apps by providing insight on where appropriate adjustments and improvements need to be made to ensure user satisfaction.

**Objective:**

A focus group with 7 participants was conducted to examine the app’s usability and collect feedback for future iterations.

**Methods:**

Past Small Steps for Big Changes clientele participated in a cognitive walkthrough of 8 novel tasks and completed the System Usability Scale survey. Participants were then given the option to use the app for 3 weeks before completing the User-Mobile Application Rating Scale.

**Results:**

Analysis of the cognitive walkthrough identified 26 usability problems; each was coded using a heuristic evaluation to describe usability errors. The most frequently coded errors included inappropriate progress feedback, information appearing in an illogical order, counterintuitive design, and issues with app aesthetics. A mean summary score of 66.8% (SD 18.91) was reported for the System Usability Scale, representing a marginal acceptability score and indicating that design issues needed to be resolved. A User-Mobile Application Rating Scale mean score of 3.59 (SD 0.33) was reported, implying an average acceptability rating.

**Conclusions:**

These findings identified necessary improvements in the app, ranging from minor aesthetic problems to major functionality problems. Involving end users allows the app to be tailored to the client’s preferences and increases the likelihood of usage. This app aligns with Small Steps for Big Changes’ program components and behavior change techniques that can improve health outcomes for future clients and allow them to self-monitor their exercise, diet, and goals.

## Introduction

### Background

Involving end users in mobile health (mHealth) technology development increases the value and practical use of the product [1,2]. Recognizing a client’s expertise through collaboration allows end users to effectively express their needs and expectations during the development phases of mHealth apps [3]. mHealth technologies have been used to increase accessibility to health care resources [4] and increase patient autonomy [5]. The uptake of mHealth technology has been shown to improve chronic disease management including improvements in weight management and hypertension, and decreased hospitalizations [6]. Within diabetes research, mHealth tools have assisted in self-management efforts, improved glycemic control [6-8], and are acceptable by users [9]. mHealth apps allow users to play an active role in their health, subsequently improving health outcomes. To capitalize on these advantages, an mHealth app was developed to supplement a diabetes prevention program run in British Columbia, Canada.

Small Steps for Big Changes is a community-based diabetes prevention program designed for individuals with prediabetes [10]. Small Steps for Big Changes is delivered in 6 sessions over 4 weeks using a client-centered counseling style known as motivational interviewing with follow-ups over 12 months. Clients work one-on-one with their coach to cover several topics including goal setting, healthy food choices, and exercise adherence. During the program’s first session, clients are introduced to self-monitoring techniques, including tracking diet and exercise. Various forms of tracking are discussed, including commercial mHealth apps, journals, paper tracking, and exercise logs. While the most prominent method of tracking used in the program is commercially available mHealth apps, several challenges have been reported by Small Steps for Big Changes clients and coaches with such tools [11]. This program-specific mHealth app was developed to supplement the Small Steps for Big Changes program and to fill the gap in mHealth apps previously identified by Small Steps for Big Changes participants. Involving end users during development supported initiatives to ensure the app was relevant and useful. This mHealth app was developed to support the Small Steps for Big Changes program by integrating the frequently used behavior change techniques throughout the 6 sessions, emphasizing goals, planning, feedback, and monitoring [12].

Development of the Small Steps for Big Changes mHealth app was guided by the FASTER (Framework for Accelerated and Systematic Technology-Based Intervention Development and Evaluation Research), a 3-phase framework that recognizes the complexities of conducting research on mHealth technologies in a timely fashion while also consistently engaging end users throughout [13]. Phase 1 included a needs assessment, literature review, and preliminary evaluation; the feedback and results from phase 1 helped inform the app prototype used in this usability study [14]. This study focused on the second phase of FASTER, examining the progressive usability of the app [13]. In this phase, small-scale usability testing helped uncover usability errors, levels of acceptability, and improvements needed before large-scale implementation (phase 3 of FASTER). Usability testing is critical for sustained use and uptake of mHealth apps by identifying where changes can be made [13]. An app that is difficult to navigate with an interface that is nonintuitive limits usage. mHealth technologies provide opportunities to self-monitor and manage health behaviors for individuals with chronic disesase [15]. Successful self-monitoring of behaviors involves consistency; if carried out on an app, this needs an interface that is easy to use and will encourage the likelihood that goals will be recorded and revisited. Understanding where usability errors exist will inform appropriate changes needed to improve app features and increase user satisfaction [13].

### Objective

The aim of this study was the answer the following questions: where do existing usability errors exist in the Small Steps for Big Changes mHealth app and is the app deemed usable by past Small Steps for Big Changes participants? To answer this question, this study used a mixed-methods approach involving a cognitive walkthrough (CW) focus group, take-home testing period, and self-reported usability surveys to engage end users in the examination of usability of a Small Steps for Big Changes mHealth app.

## Methods

### Small Steps for Big Changes App Development

Pathverse, the app-building company used for this project that specializes in no-code app building for research and learning [16], worked with our research team to create a platform specific to the needs of the Small Steps for Big Changes program. Several meetings took place with Pathverse and the research team to organize content, create select features, and tailor the content to the Small Steps for Big Changes program. This app included basic self-monitoring features, including tracking for steps, minutes of daily exercise, weight, waist circumference, and hemoglobin A_1c_ (HbA_1c_). Additional capabilities included logging exercise sessions, recording goals, diary entry opportunities, and access to additional frequently asked questions and resources including nutritional cooking videos and program content. Additional details on layout and design can be seen in the attached screenshots available in Multimedia Appendix 1.

### Participants

A total of 7 participants were recruited to participate in this study. A sample size between 6 and 10 participants was determined a priori based on past literature exploring user-centered CW methods in mHealth chronic disease self-management [17,18]. Secondary measures used to collect additional usability data included the System Usability Scale (SUS) [19,20] and User-Mobile Application Rating Scale (uMARS) [21]. Past Small Steps for Big Changes clients who had completed the 4-week training phase of the program were invited to take part in this study. Eligible participants included past Small Steps for Big Changes clients who were adults aged 18 years or older, able to read and write in English, assessed for prediabetes by one of the following means: (1) physician-diagnosed prediabetes, (2) HbA_1c_ values between 5.7% and 6.4%, and (3) an American Diabetes Association risk questionnaire score indicating increased risk of diabetes (>5). Participants also needed to have access to a mobile device with internet access (such as a tablet or smartphone). In an attempt to represent the current demographic of the Small Steps for Big Changes program (75% of individuals identified as female and 25% as male), participants were purposively sampled. Participants were asked to bring an internet-enabled mobile device during their laboratory visit with capabilities to connect and access the device over the 3-week take-home period.

### Ethical Considerations

Informed consent was obtained from all individual participants and all procedures performed were in accordance with the ethical standards of the University of British Columbia Behavioural Research Ethics Board (H22-01399).

### CW: Phase 1

Participants were invited to engage in a CW focus group at the University of British Columbia Okanagan Campus. CWs involve a task-based method to explore the usability of digital health technologies and when used in conjunction with questionnaires are part of a multistep process to evaluate usability [17,18,22]. As participants arrived, research assistants helped to provide assistance to connect to the internet, download the app, and create a user profile. A 10-minute demonstration of the Small Steps for Big Changes app on a large projector screen was presented before the CW began to account for the app’s novelty. During this demonstration a researcher lead a familiarization walkthrough of each of the function landing pages (home page, tracking page, and resources page) as well as some of the button functions (go-back, save, and delete). Participants followed along on their devices and were given time to play with navigating the app before starting the novel tasks. After this period, each participant was provided with a task booklet outlining 8 novel tasks to be completed during the CW focus group. Each task had 3 corresponding questions used to identify usability problems about the task and assist with the facilitation of group discussion. The workbook was designed for additional observations to be recorded during each task. During the CW, participants were asked to rotate through leading the completion of a task using their instincts by informing the facilitator how to perform the task on a large screen for the group to follow. After each task, all participants were asked to record their observations and share their thoughts about any usability problems they encountered. Each participant lead at least 1 task during the CW. Once all tasks were completed in the CW, participants were granted access to the app for an additional 3 weeks to use it as they pleased, simulating a real-world setting. During this time, participants were encouraged to make note of any additional usability errors, feedback for future modifications, and general reactions or evaluations. This information was provided along with compensation following completion of this study.

### Mobile Device Proficiency Questionnaire and Demographics Survey

The Mobile Device Proficiency Questionnaire (MDPQ-16) [23] was used to assess participants’ mobile device proficiency, and demographics were collected for descriptive reporting. Participants self-reported their age, sex, gender, whether they were born in Canada, whether they identify as Indigenous or a visible minority, their highest level of education completed, occupational status, household income, and marital status. During the initial laboratory visit, participants were asked to complete a demographics survey and the MDPQ-16 on iPads (Apple Inc) before beginning the CW. The MDPQ-16 includes 16 questions organized into 8 subscales such as mobile device basics, communication, internet, troubleshooting, and are answered on a 5-point scale (1=never tried and 5=tried very easily). Each subscale score is averaged and then summed, with a maximum score of 40 (a higher score indicates greater proficiency [23]).

### About SUS

After completing the CW tasks, participants were asked to complete the SUS [19]. The SUS was used to assess system usability. This brief 10-question survey asks general usability questions about ease of use and complexity on a 5 point-scale (1=strongly disagree and 5=strongly agree). The SUS is a widely used scale, a recent meta-analysis concluded that the SUS was a suitable measure for evaluating the usability of digital health apps [20]. Final SUS scores range from 0‐100, with higher scores indicating better usability [19] with a benchmark acceptability score of 68 [20]. This survey was delivered immediately after the CW to each participant on an iPad.

### User Version of the Mobile App Rating Scale: Phase 2

After completing an optional take home period, the uMARS [21] was used to assess the quality and usability of the mHealth app. The uMARS items are answered on a 5-point scale, representing the following 1-inadequate, 2-poor, 3-acceptable, 4-good, and 5-excellent [21]. The uMARS is a reliable tool to measure app quality and has been used to assess mHealth apps including self-management behaviors associated with type 2 diabetes [24]. This survey offered additional detail and depth compared to the SUS and was delivered to the participants after an optional 3-week at-home phase. The uMARS consists of 4 subscales focused on engagement, functionality, aesthetics and information. A mean score for each subscale is calculated, and a total app quality score is calculated by averaging the subscale means. The survey also includes additional questions about app subjective quality, perceived impact, and an option to leave further written comments. Participants were given information about the take-home phase of this study and were asked to use the app freely for 3 weeks. After this take-home period, participants were sent a survey link to the uMARS.

### Analysis

All survey data from the demographics, MDPQ-16, SUS, and uMARS were descriptively analyzed using SPSS (version 27; IBM Corp) to examine mean scores, SD, and range. The CW was analyzed through an audio recording and transcript. The usability problems identified in the CW task workbook and transcript were coded using Nielsen’s heuristic evaluation [25,26]. The 10 usability heuristics outline general principles for user interface design; if an identified usability problem violates any of the 10 heuristics, it is coded accordingly. Furthermore, 2 coders completed the qualitative analysis and assigned severity scores to each of the usability errors identified.

## Results

### Demographics and MDPQ-16

A total of 5 of the 7 participants were aged older than 70 years, and 2 were between 55 and 65 years of age. No individuals identified as Indigenous or a visible minority. Further, 2 participants identified as having a disability. There was variance in education level and family income; 5 participants were retired. Table 1 reports on more detailed demographic results. MDPQ-16 scores were reported to assess levels of mobile device proficiency. The mean (SD) MDPQ-16 total score was 36.36 and SD of 3.42, individual MDPQ-16 scores are reported in Multimedia Appendix 1. These MDPQ-16 scores represented high levels of mobile device proficiency for our participants compared to those identified in previous literature [23].

**Table 1.** Demographic characteristics of participants.

Sample characteristic	Participants who selected this response
Age (years), mean (SD)	70 (7.47)
**Sex, n (%)**	
Female	4 (57)
Male	3 (43)
**Gender, n (%)**	
Woman	4 (57)
Man	3 (43)
**Born in Canada, n (%)**	
Yes	4 (57)
No	3 (43)
No	7 (100)
No	7 (100)
**Identifies as a person with a disability, n (%)**	
Yes	2 (29)
No	4 (57)
Prefer not to answer	1 (14)
**Level of education, n (%)**	
High school	1 (14)
University certificate or diploma below the bachelor level	3 (43)
Postgraduate degree	2 (29)
College, CEGEP^a^, or other nonuniversity certificate or diploma	1 (14)
**Working status, n (%)**	
Working full time	2 (29)
Retired	5 (71)
**Annual income level (US $)**	
$41,688 to $62,531	1 (14)
$62,532 to $82,681	1 (14)
$104,220 or more	3 (43)
Prefer not to answer	2 (29)
**Marital status, n (%)**	
Married	6 (86)
Widowed	1 (14)

a CEGEP, Collège d'enseignement general et professionnel, which means general and professional teaching college

### About CW

The CW lasted 60 minutes in duration. Participants were asked to complete 8 novel tasks while engaging with the Small Steps for Big Changes app. A total of 26 usability problems were identified and assigned severity scores in the recording transcript and client workbooks. The highest number of usability problems identified for a specific task was 7 for task 1, and the lowest was 0 for task 8. All severity scores and usability problems identified for each task and corresponding Nielsen heuristics are reported in Table 2.

**Table 2.** Usability problems identified in associated workbook task completion.

Task	Number of usability problems identified	Mean severity score (SD)	Taps expected to complete task (n)	Actual taps to complete task (n)	Nielsen heuristics identified (*f*)
1-Record a weight value	7	2.1 (0.69)	5	8	1 (3), 2 (4), 3 (1), 4 (2), 6 (4), 8 (2)
2-Navigate to the FAQ^a^ page	2	0.4 (0.53)	4	4	3 (1), 6 (1), 8 (1)
3-Log an exercise	3	1 (1.15)	17	18	1 (1), 2 (1), 4 (1), 6 (2), 8 (2)
4-Create a diary entry	1	0.5 (0.58)	7	7	10 (1)
5-Record a goal	5	1.9 (1.38)	12	15	1 (1), 2 (2), 3 (1), 4 (1), 5 (1), 6 (2), 9 (1)
6-Navigate to a module	4	1.6 (0.53)	6	9	2 (3), 3 (1), 8 (1), 10 (2)
7-Update the hemoglobin A_1c_ value within the tracking page	4	0 (0)	10	10	2 (1), 4 (2), 6 (2), 8 (1)
8-Navigate to a video resource	0	0 (0)	5	5	0

a FAQ: frequently asked questions.

The usability problems identified in the CW included issues with icon and text sizes, scroll sensitivity, system preferences, and logical location of features and information. Common system preference issues were challenges saving and editing goals and the exclusion of a 24-hour clock. Participants also had difficulty scrolling and reported favorability for a text box for selecting values. Usability problems were analyzed by coding each into 10 categories. The 26 usability problems highlighted errors associated with information appearing in an illogical order and lack of visibility of information. For example, the usability error named “tracker alignment” indicates that the weight tracking feature’s dates did not match what was seen on the graph displayed in the participant’s progress summary. This was coded as 1 (incorrect feedback provided by the system) and 2 (information appearing in an illogical order). All usability problems were coded accordingly; some were assigned only 1 Nielsen heuristic, while others were assigned multiple.

NG and KB double-coded the 26 identified usability errors according to heuristic evaluation. Nielsen heuristics of 2 (match between system and the real world) and 6 (recognition rather than recall) were the most frequently coded (11 times each), while heuristics of 5 (error prevention), 7 (flexibility and efficiency of use), and 9 (help users recognize, diagnose, and recover from errors) were rarely coded. Table 3 outlines each heuristic score, their coded frequency, and the corresponding percentage of the 49 total codes used.

**Table 3.** Nielsen heuristic frequency for usability problem codes from a total of 26 usability problems and 49 total codes.

Nielsen heuristic	Frequency (%^a^)
Visibility of system status	5 (10)
Match between system and the real world	11 (22)
User control and freedom	4 (8)
Consistency and standards	6 (12)
Error prevention	1 (2)
Recognition rather than recall	11 (22)
Flexibility and efficiency of use	0 (0)
Aesthetic and minimalist design	7 (14)
Help users recognize, diagnose, and recover from errors	1 (2)
Help and documentation	3 (6)

a %: percentage of the total frequency of codes.

### About SUS

The mean SUS score was 66.8% for the sample, with an SD of 18.91 and a large range from 50 to 97.5. Further, 4 participants rated the usability moderately low, between 50% and 60%, while the remaining 3 rated the usability highly, resulting in scores above 85%.

### About uMARS

In total, 5 of the 7 clients who participated in the CW completed the full uMARS. The app quality total score was calculated to be a mean score of 3.59/5 with an SD of 0.33, with the highest-rated subcategory being aesthetics at a mean score of 3.8 (SD 0.51) and the lowest being engagement at 3.12 (SD 0.33). The highest perceived impact scores were behavior change and awareness, both receiving a mean score of 3.33 (SD 1.03), with knowledge being the lowest at a mean of 2.8 (SD 0.84). Additional feedback for improvements included adding analytics for the progress made on the app, adding synchronizing capabilities with smartwatches to avoid manually recording physical activity and including tracking standards for specific age groups and sex. A preference to the Fitbit app was also reported by 2 participants during discussion of additional feedback after the CW. Positive comments resulting from completion of the uMARs included comments about the potential for the app to be a very useful tool, the value of recording anthropometrics such as weight and HbA_1c_, the emphasis on diabetes, and the use of the graphs as a visual representation of the client’s progress. Survey scores for usability data are reported in Table 4, and additional results from the usability surveys are included in Multimedia Appendix 1.

**Table 4.** Usability scores for quantitative surveys (N=18).

Survey	Mean (SD)		Range
MDPQ-16^a^	36.36/40 (3.42)		31‐40
SUS^b^	66.8/100 (18.91)		50‐97.5
uMARS^c^	3.59/5 (0.33)		3.03‐3.83

a MDPQ-16: Mobile Device Proficiency Questionnaire.

b SUS: System Usability Scale.

c uMARS: User-Mobile Application Rating Scale.

### Follow-Up With Pathverse

Once the research team noted the usability problems and feedback, 2 follow-up meetings were conducted with Pathverse to address the usability concerns and make appropriate improvements. Updates made by the Pathverse team included correcting minor bugs with diary notification scheduling, fixing the scaling of images and icons, and bringing consistency across devices (Samsung vs Apple). Additions made to the app included 24-hour time when inputting goals and tracking, opportunity to input manual steps, and updates for some of the language and icons used (the menu symbol, save button, and trash icon).

## Discussion

### Findings

The 26 usability problems identified in the CW identified relevant usability errors seen during task-based discussion within the group of participants. Further, 9 of the 10 Nielsen heuristics were coded, indicating usability errors across multiple domains of the app; however, most usability problems were addressed for preference changes and minor updates in the most recent iteration of the app. Other suggestions, including a hover function to show a description of functions within the app, and the ability to link the app with a Fitbit have not yet been addressed due to cost, time, and practicality implications.

The usability score of 66.8 for the SUS represented a marginal usability score [19,20]. The variance could be due to a range in expectations of the app and its functions; a few users expressed they had been using different health monitoring apps, which they were quite satisfied with, which may have influenced their perceptions of the Small Steps for Big Changes app. After 3 weeks of optional use, a uMARS mean score of 3.59 was reported, describing an acceptable score [22]. MDPQ-16 scores were relatively high in our sample; we can have confidence that the feedback provided was independent of a lack of skills or ability to use mobile apps. Within our sample, moderate levels of usability may have been due to a low level of willingness to track in general. We did not purposively sample those who favored smartphone tracking over other methods such as tracking in a journal or workbook,; and some users may have had little interest in tracking at all.

The literature has identified perceived usability as an existing barrier to mHealth usage [27]; this aligns with the moderate levels of usability reported in this study. However, high levels of usability have been reported by select mHealth technologies designed for individuals with type 2 diabetes [9,28], which counter the moderate levels of usability seen in this study. Although the levels of usability have demonstrated mixed results, when comparing user feedback, various themes did align with our findings. Users agreed on noting similar improvements, including adding more knowledge and information available on the mHealth apps [28], suggesting that mHealth app content may be oversimplified. Interestingly, this study reported high levels of mobile device proficiency from adults with a mean age of 70 years, which may contradict previous research noting older age as a barrier to mHealth usage [27].

This study identified usability problems and necessary improvements for the Small Steps for Big Changes program app. With only moderate levels of usability reported, these results emphasize the need for user feedback during the development process. This highlights the importance of the end user’s involvement in app development and prototype stages. Several iterations of mHealth technology should be tested before large-scale implementation to ensure the end product is relevant and highly useful to users. This study demonstrates that early usability evaluations can help identify and address issues at the beginning of the development cycle, saving time and resources in the long run. The iterative nature of this study’s approach, where usability problems were identified and subsequently addressed, underscores the importance of continuous improvement. Following the steps of the FASTER framework helped ensure that necessary steps were not overlooked; during this process, essential findings for decision-making can save on cost and the long-term success of an mHealth app [13]. This study highlights the importance of tailoring mobile apps, especially health-related ones, to meet the specific needs and preferences of older adults. Developers should prioritize user-centered design principles, considering factors such as font size, icon clarity, and ease of navigation to enhance usability for this demographic.

Past research has highlighted the importance of integrating multiple methods to assess usability in digital health evaluation [22],; this study incorporated various modes of feedback through a CW, real-world use for 3 weeks, validated questionnaires, and qualitative feedback. Diverse feedback sources provide a more comprehensive understanding of usability and user experiences. Each feedback method uncovers different aspects of usability. Combining various feedback sources allows for a holistic evaluation of the mobile app. This approach ensures that usability problems are not only identified but also validated across different dimensions. It helps prioritize issues that have the most significant impact on users. The real-world use over 3 weeks provides insights into how usability evolves over time. It helps identify whether initial issues persist or if users adapt to the app. The combined feedback informs tailored iterations. Specific issues can be addressed when identified through different sources in a targeted manner. For example, usability problems identified in CW may lead to design changes, while questionnaire data can guide overall satisfaction improvements.

### Limitations

Limited diversity within our sample was seen, specifically for those identifying as Indigenous or a visible minority. While this is representative of the current demographics served by Small Steps for Big Changes, as Small Steps for Big Changes expands to more communities, the potential to target more diverse opinions should be highlighted, especially for those who may benefit the most from increased accessibility (eg, individuals living in rural communities). This study did not explore long-term user compliance and security issues; further research and considerations should be explored to identify solutions around these limiting factors [29]. Considerations including (1) a small sample size when reporting uMARS results and (2) an unrealistic simulation of app implementation (providing assistance during installation and a familiarization of the app) should be taken into account when interpreting the results.

### Future Directions for Research

Future research may look toward phase 3 of FASTER through large-scale implementation and evaluation of the app as a supplement to the Small Steps for Big Changes diabetes prevention program to examine the app’s effectiveness in improving health behaviors and clinical outcomes for clients. mHealth prompts such as texts and push notifications have been well-received by users in diabetes prevention [30]. Looking toward these capabilities within the Small Steps for Big Changes app is an additional function that may benefit client health outcomes by allowing them to self-monitor their exercise, diet, and goals. Future iterations of the app will call for additional cycles of usability testing and evaluation to ensure a satisfactory user experience is reached. Usability is an important aspect of user experience; however, additional factors including incentivizing, convenience, and practicality of mHealth apps may also influence decisions to integrate their use in practice. For example, the results of this study report that the lowest category in the uMARS scale was engagement. In the uMARS, user engagement refers to whether the app is entertaining, interesting, customizable and interactive; if users do not find the app engaging they may be less incentivized to use it.

### Conclusions

Diabetes self-management efforts can be improved using mHealth technologies; it can help establish meaningful routines, allow clients to better understand their health conditions and status, and provide additional outlets for interactions with health care professionals and resources [31]. Providing tools to make behavior change efforts easier for clients will lead to better outcomes; however, these tools need to be well-accepted by users to increase the likelihood of usage. This study successfully assessed the usability of a self-monitoring app for individuals with prediabetes by identifying 26 usability errors and recognizing where improvements needed to be made. These activities are necessary for generating research evidence for technology-based interventions [13] to ensure usage is not a limiting factor. Moderate usability levels were reported by users, prompting a new iteration of the app, emphasizing the importance of user involvement. Involving end users in this process allows the mHealth app usage experts to make decisions during development. Overlooking usability testing during applicaton development can result in premature large-scale implementation before critical changes and errors can be addressed. Embracing multiple feedback modes reflects a user-centric approach to design and evaluation. It demonstrates a commitment to understanding the user experience from various angles, which is essential for creating user-friendly apps.

## Supplementary material

10.2196/59386 Multimedia Appendix 1 Additional usability data and screenshots of app platform.

## Abbreviations

CW: cognitive walkthrough
FASTER: Framework for Accelerated and Systematic Technology-Based Intervention Development and Evaluation Research
HbA_1c_: hemoglobin A_1c_
MDPQ-16: Mobile Device Proficiency Questionnaire
mHealth: mobile health
SUS: System Usability Scale
uMARS: User-Mobile Application Rating Scale
