# Supplementary material for: Involving End Users in the Development and Usability Testing of a Smartphone App Designed for Individuals With Prediabetes: Mixed-Methods Focus Group Study
Source: JMIR Form Res. 2025 Feb 11;9:e59386. doi: 10.2196/59386 (PMC11835782; doi:10.2196/59386)

Summary of MDPQ-16 scores

| Scale | N | M | SD | Range |
| --- | --- | --- | --- | --- |
| Mobile Device Basics | 7 | 4.86 | 0.38 | (4.00-5.00) |
| Communication | 7 | 4.86 | 0.24 | (4.50-5.00) |
| Data & File Storage | 7 | 3.79 | 0.91 | (3.00-5.00) |
| Internet | 7 | 4.86 | 0.38 | (4.00-5.00) |
| Calendar | 7 | 4.71 | 0.76 | (3.00-5.00) |
| Entertainment | 7 | 4.36 | 0.75 | (3.00-5.00) |
| Privacy | 7 | 4.29 | 0.70 | (3.50-5.00) |
| Troubleshooting and Software Management | 7 | 4.64 | 0.48 | (4.00-5.00) |
| MDPQ-16 Total | 7 | **36.36/40** | **3.42** | **(31.00-40.00)** |

Summary of SUS scores

| Individual | SUS Score (%) |
| --- | --- |
| Participant 1 | 97.5 |
| Participant 2 | 70 |
| Participant 3 | 55 |
| Participant 4 | 55 |
| Participant 5 | 52.5 |
| Participant 6 | 50 |
| Participant 7 | 87.5 |
| Mean, SD, Range | **66.8%**, 18.91, (50-97.5) |

Summary of UMARS scores

| Scale | N | Mean | SD | Range |
| --- | --- | --- | --- | --- |
| Engagement | 5 | 3.12 | 0.33 | (2.60-3.40) |
| Functionality | 5 | 3.70 | 0.33 | (3.25-4.00) |
| Ascetics | 5 | 3.80 | 0.51 | (3.00-4.33) |
| Information | 5 | 3.75 | 0.53 | (3.00-4.25) |
| **APP QUALTITY TOTAL SCORE** | **5** | **3.59** | **0.33** | **(3.03-3.83)** |
| Subjective App Quality | **6** | **3.08** | **0.58** | **(2.50-4.00)** |

| Perceived Impact on: | N | Mean Rating | SD | Range |
| --- | --- | --- | --- | --- |
| Awareness | 6 | 3.33 | 1.03 | 2-5 |
| Knowledge | 5 | 2.80 | 0.84 | 2-4 |
| Attitudes | 6 | 3.17 | 1.17 | 2-5 |
| Intentions to change | 5 | 3.00 | 1.23 | 2-5 |
| Help seeking (if needed) | 6 | 3.17 | 1.17 | 2-5 |
| Behaviour change | 6 | 3.33 | 1.03 | 2-5 |
| Further comments? | 4 | The one thing that I find missing is the link to the fitbit for a start - here we are collecting all sorts of health data and unless put into the App manually it does not get there. There need to be some analytics which provide feedback on progress being made. Somehow the app needs to be more inviting and provide some commentary and not just a repository for information. It has the possibility of being a very useful tool.  I feel this app would be of more use to me if I were able to synchronize it with a step counter or something like that (Fitbit etc). I found it easy to forget to track my activities and times and manually record them. The record of weight, A1c etc is good. I can track my progress with the graph quite easily. In all, with the exception of the focus on diabetes, as it is now, is quite an average app, similar to many on the market.  The inclusion of age/gender standards would be of value.  I think the FitBit app gives more and better data to manage health issues. | | |


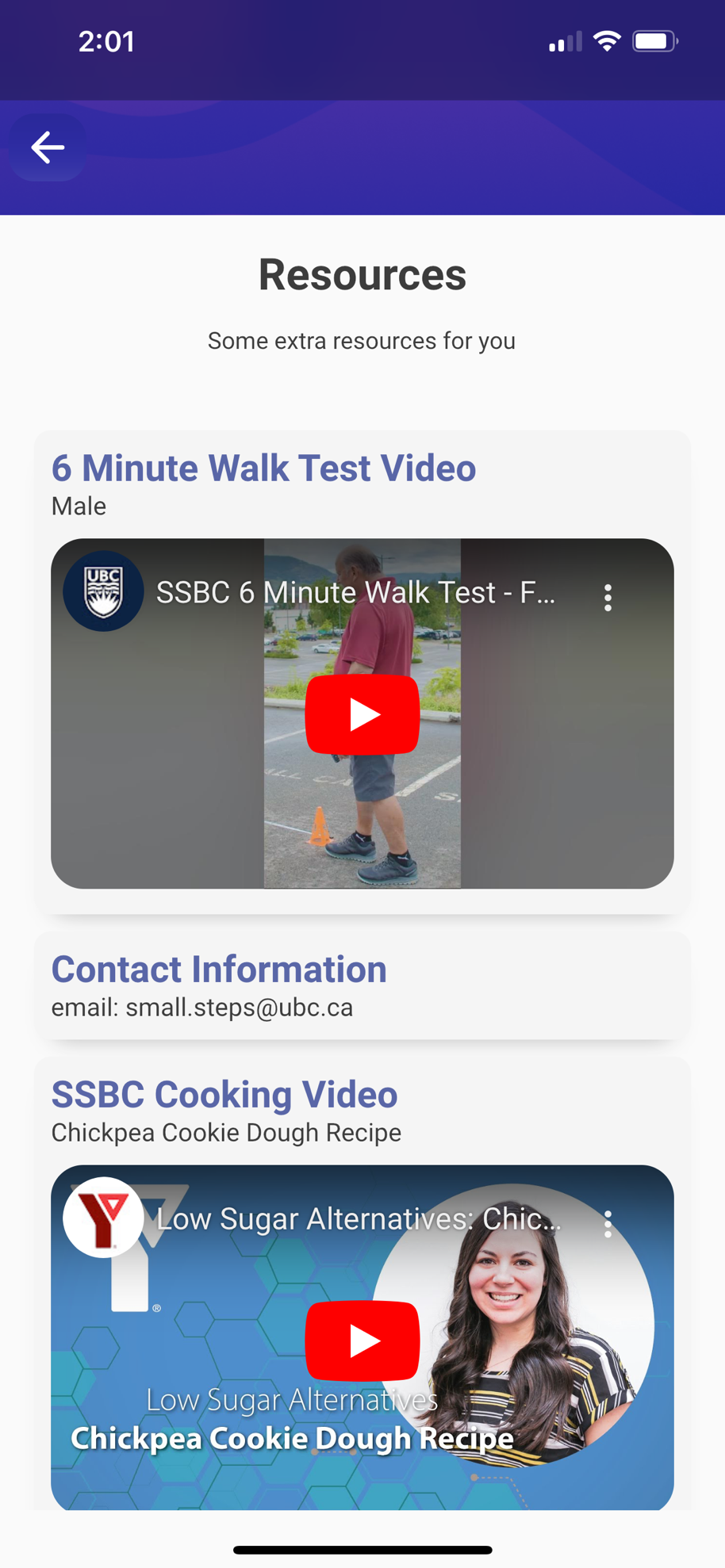

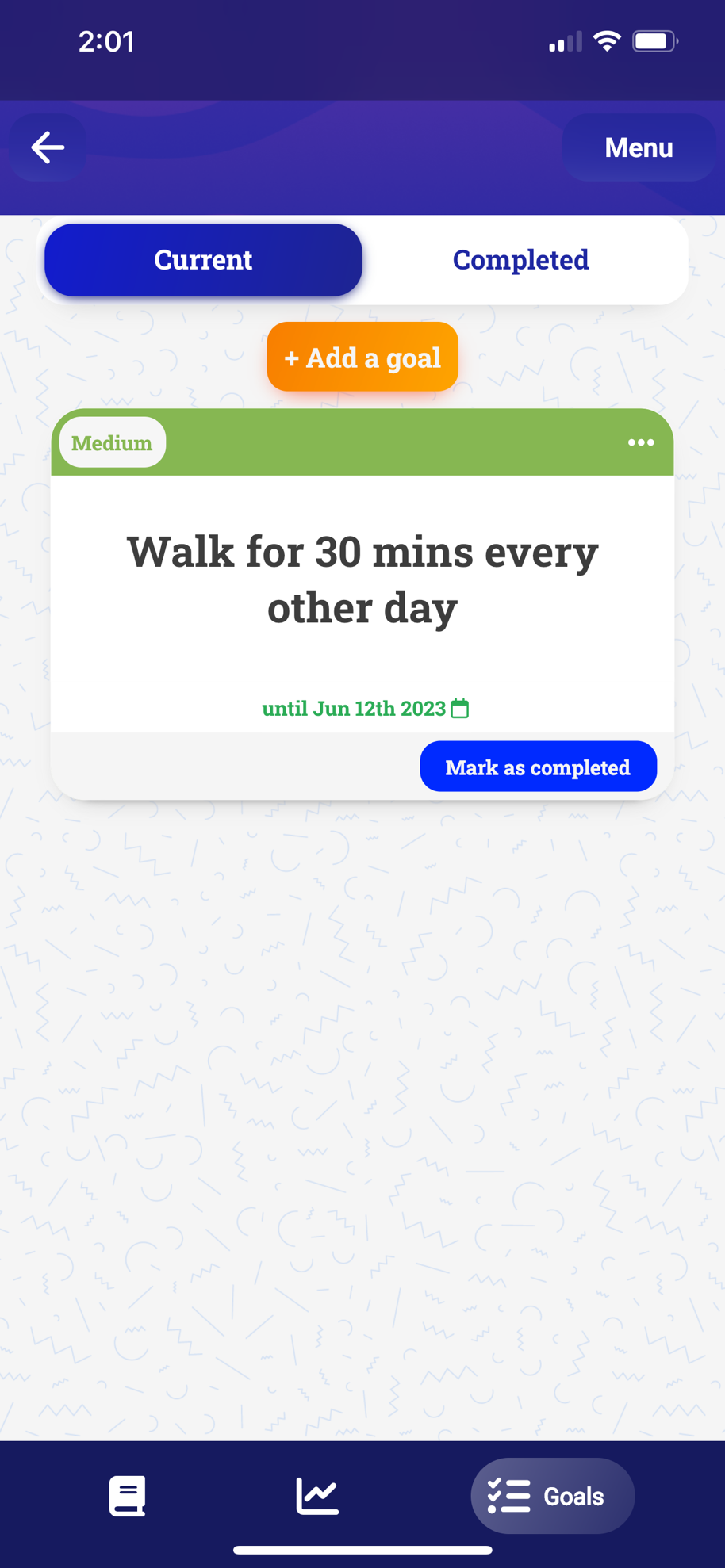

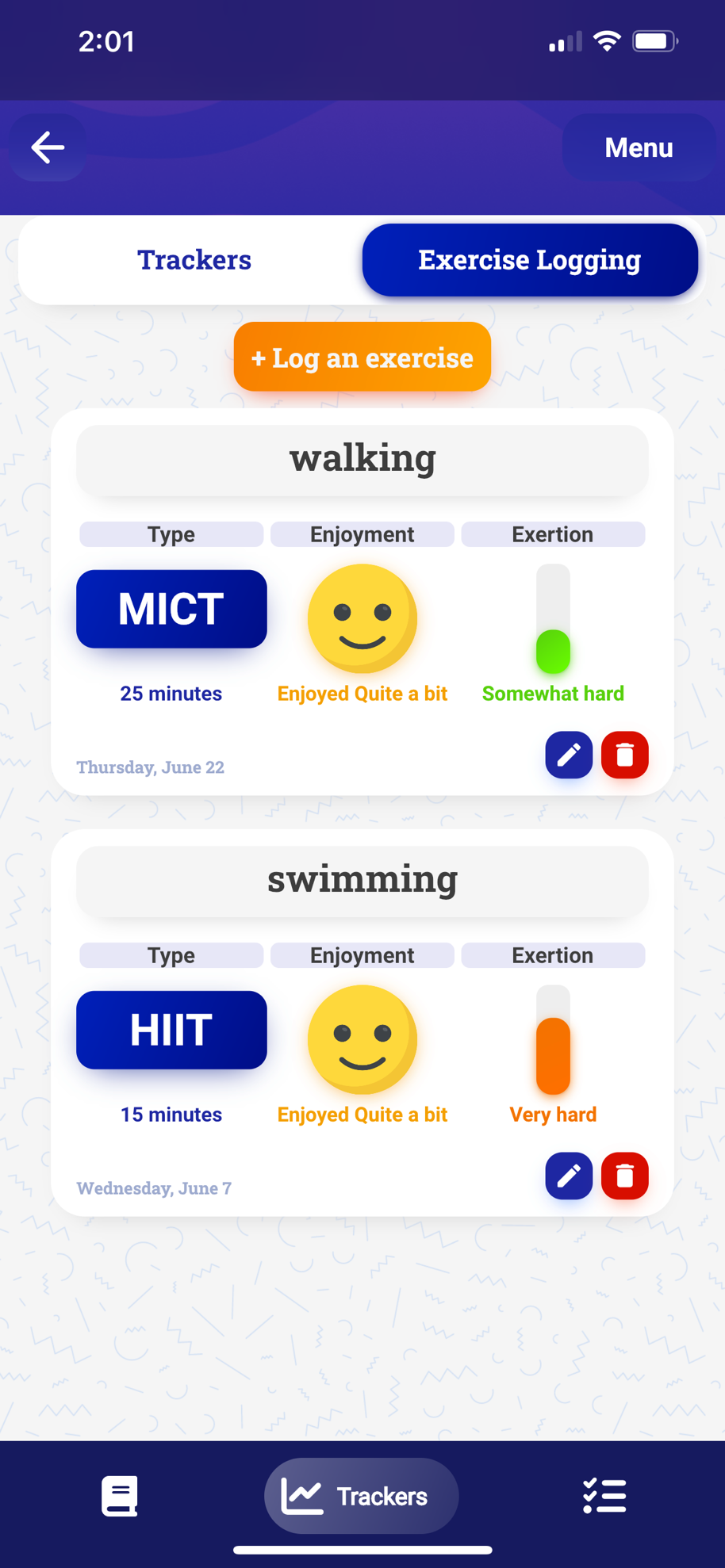

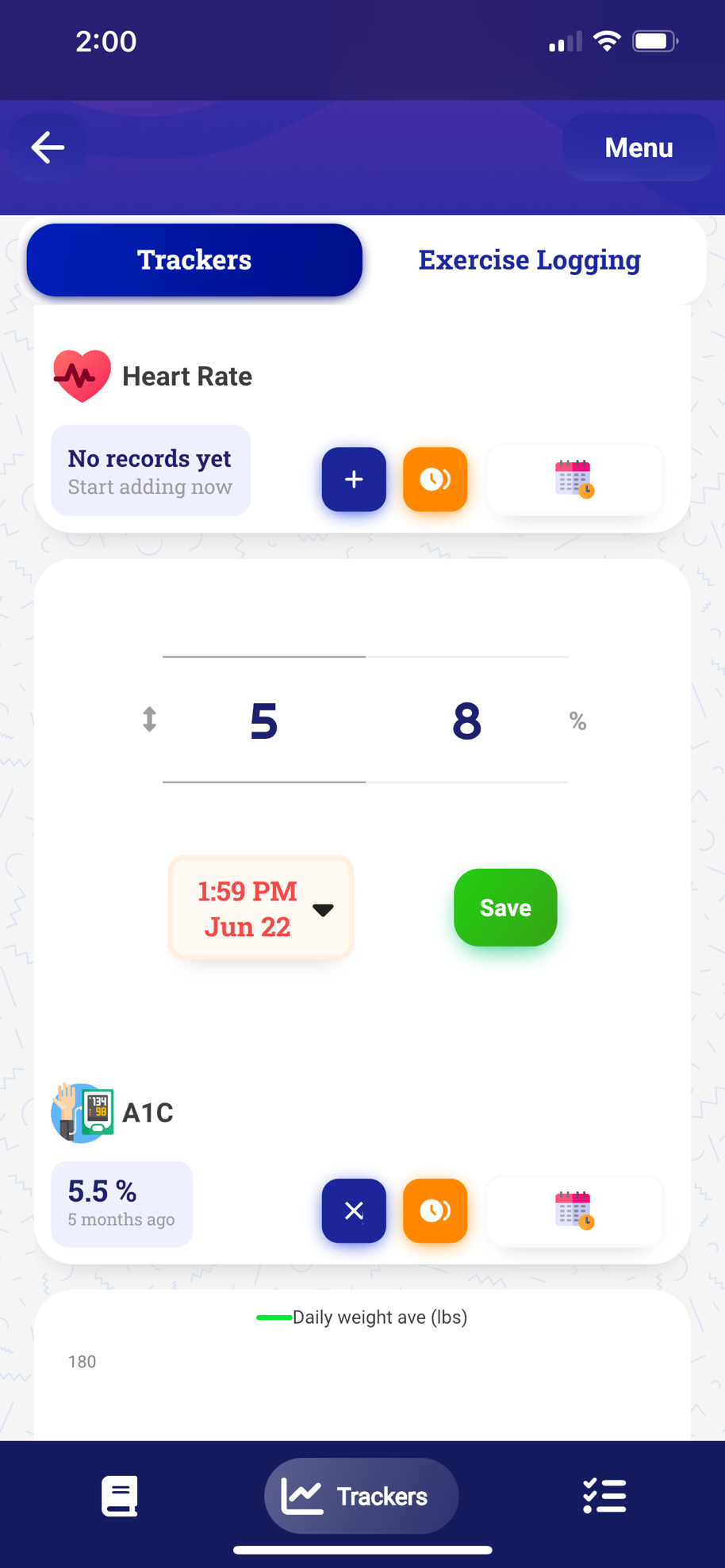

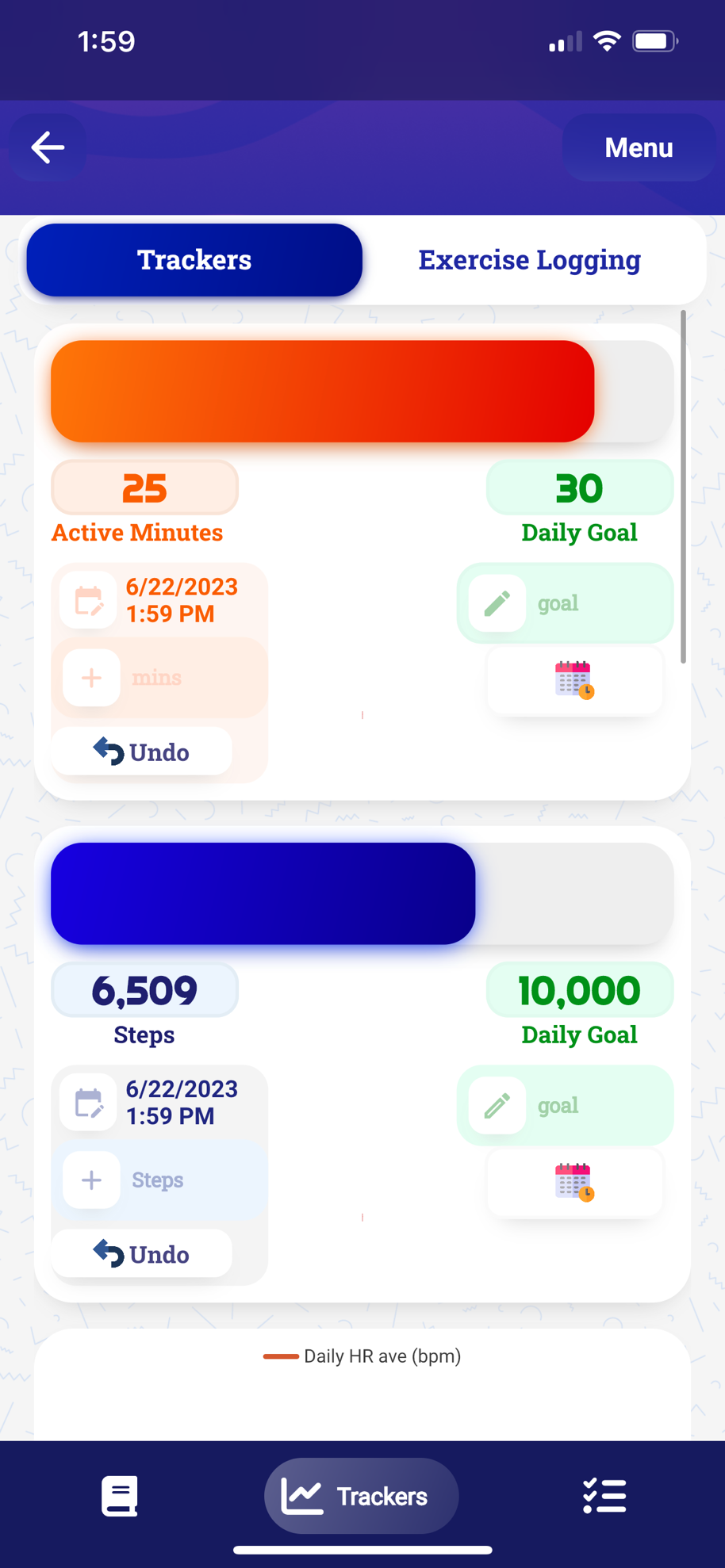

Supplement: Multimedia Appendix 1 [file formative-v9-e59386-s001.docx]
